# Blood DNA methylation marks discriminate Chagas cardiomyopathy disease clinical forms

**DOI:** 10.3389/fimmu.2022.1020572

**Published:** 2022-09-29

**Authors:** Pauline Brochet, Barbara Ianni, João P. S. Nunes, Amanda F. Frade, Priscila C. Teixeira, Charles Mady, Ludmila R. P. Ferreira, Andreia Kuramoto, Cristina W. Pissetti, Bruno Saba, Darlan D. S. Cândido, Fabrício Dias, Marcelo Sampaio, José A. Marin-Neto, Abílio Fragata, Ricardo C .F. Zaniratto, Sergio Siqueira, Giselle D. L. Peixoto, Vagner O. C. Rigaud, Paula Buck, Rafael R. Almeida, Hui Tzu Lin-Wang, André Schmidt, Martino Martinelli, Mario H. Hirata, Eduardo Donadi, Virmondes Rodrigues Junior, Alexandre C. Pereira, Jorge Kalil, Lionel Spinelli, Edecio Cunha-Neto, Christophe Chevillard

**Affiliations:** ^1^ Aix Marseille Univ, TAGC Theories and Approaches of Genomic Complexity, Institut MarMaRa, Marseille, France; ^2^ Laboratory of Immunology, Heart Institute Instituto do Coração(InCor), School of Medicine, University of São Paulo, São Paulo, Brazil; ^3^ Division of Clinical Immunology and Allergy, School of Medicine, University of São Paulo, São Paulo, Brazil; ^4^ Instituto Nacional de Ciência e Tecnologia, INCT, iii- Institute for Investigation in Immunology, São Paulo, Brazil; ^5^ Myocardiopathies and Aortic Diseases Unit, Heart Institute, Instituto do Coração (InCor), School of Medicine, University of São Paulo, São Paulo, Brazil; ^6^ RNA Systems Biology Laboratory (RSBL), Departamento de Morfologia, Instituto de Ciências Biológicas, Universidade Federal de Minas Gerais, Belo Horizonte, Minas Gerais, Brazil; ^7^ Laboratory of Immunology, Universidade Federal Do Triângulo Mineiro (UFTM), Uberaba, Brazil; ^8^ Laboratório de Investigação Molecular em Cardiologia, Instituto de Cardiologia Dante Pazzanese (IDPC), São Paulo, Brazil; ^9^ School of Medicine of Ribeirão Preto (FMRP), University of São Paulo, Ribeirão Preto, Brazil; ^10^ Pacemaker Clinic, Heart Institute Instituto do Coração (InCor), School of Medicine, University of São Paulo, São Paulo, Brazil; ^11^ Heart Institute Instituto do Coração (InCor), School of Medicine, University of São Paulo, São Paulo, São Paulo, Brazil; ^12^ Department of Clinical and Toxicological Analyses, Faculty of Pharmaceutical Sciences, University of São Paulo (USP), São Paulo, Brazil

**Keywords:** chagas disease, cardiomyopathy, blood, biomarkers, methylation

## Abstract

Chagas disease is a parasitic disease from South America, affecting around 7 million people worldwide. Decades after the infection, 30% of people develop chronic forms, including Chronic Chagas Cardiomyopathy (CCC), for which no treatment exists. Two stages characterized this form: the moderate form, characterized by a heart ejection fraction (EF) ≥ 0.4, and the severe form, associated to an EF < 0.4. We propose two sets of DNA methylation biomarkers which can predict in blood CCC occurrence, and CCC stage. This analysis, based on machine learning algorithms, makes predictions with more than 95% accuracy in a test cohort. Beyond their predictive capacity, these CpGs are located near genes involved in the immune response, the nervous system, ion transport or ATP synthesis, pathways known to be deregulated in CCCs. Among these genes, some are also differentially expressed in heart tissues. Interestingly, the CpGs of interest are tagged to genes mainly involved in nervous and ionic processes. Given the close link between methylation and gene expression, these lists of CpGs promise to be not only good biomarkers, but also good indicators of key elements in the development of this pathology.

## Introduction

Chagas disease is an endemic disease from South America, caused by a parasite, *Trypanosoma cruzi*, and affecting around 7 million people. With migration flow, this disease can now be found in non-endemic country, notably in North America (1) (n > 300,000), Europe (2) (n > 100,000), Japan (3) (n > 4,000) or Australia (4) (n > 1,000). After the infection, patients present an acute stage which is mostly asymptomatic (ASY). Then comes the chronic forms, where 70% of them remains asymptomatic, with no end organ damage (the so-called indeterminate stage). However, 30% develop Chagas disease Cardiomyopathy (CCC) (5). CCC had been divided in two stages based on heart ejection fraction: moderate CCC (EF ‗ 0.4) and severe CCC (EF < 0.4) (6–8). Some drugs are effective on *T. cruzi*, but does not cure the CCC, reducing the parasitemia, without having any effect on heart damage (9). The only way out for CCC patients is the placement of a pacemaker, or a heart transplant. The early diagnosis of Chagas disease is therefore essential.

During the acute stage, disease diagnosis is commonly made by microscopy, considering the limited sensitivity of the direct test (10). However, in chronic stage, parasitemia is very low, or even null. The Pan American Health Organization (PAHO) recommends using two serological tests (two techniques based on different antigens) in parallel and, in case of discordant results, to perform these tests again on a new sample (11). If the results remain unclear, a confirmation test should be achieved (12). For CCC especially, an ECG and/or an echocardiogram is made to confirm cardiac involvement (13). BNP and NT-proBNP, well-known markers be associated with cardiac dysfunction (14), have been associated to Chagas cardiomyopathy (15, 16), but are not specific to this pathology. Others markers, including miRNAs (17, 18), cytokines (19) or metalloproteinases (20) have been proposed as biomarkers for CCC, but no confirmation has been made in a test cohort at this time. The only diagnosis of CCC currently in place is a clinical diagnosis, which is difficult to access for the most remote populations.

A previous analysis (21) has highlighted differences of DNA methylation in blood of asymptomatic and CCC patients. Moreover, some differences have also been demonstrated between moderate and severe CCC. Blood DNA methylation has already been proposed as biomarker for several diseases (22–24). Here, we used machine learning methods on both asymptomatic and CCC blood DNA methylation data to predict Chagas disease, as well as Chagas disease stage.

## Methods

### Ethical considerations

The protocol was approved by the institutional review boards of the University of São Paulo School of Medicine and INSERM (French National Institute of Health and Medical Research). Written informed consent was obtained from all patients. All experimental methods comply with the Helsinki Declaration.

### Blood DNA collection and DNA methylation analysis

Blood samples (5 to 15 ml of blood) from CCC patients were collected in EDTA tubes. Genomic DNA was isolated using standard salted methods and the methylation analysis was done using the same protocol as tissue DNAs.

### Blood DNA methylation data

138 patients were selected randomly from our Chagas bank. It included 48 asymptomatic subjects, 46 moderate CCC patients and 44 severe CCC patients (**Supplementary Table 1**). The age and sex ratio were not significantly different between the 3 groups (age mean and ratio female/male for all phenotypes: asymptomatic: age: 57.63, ratio =1; moderate: age: 56.89, ratio=1.14; severe: age: 59.59, ratio=0.95). In a second time, these 138 samples were randomly distributed between the training (70%) and validation (30%) cohorts. This random distribution was done in such a way that the age and sex ratio was still not so different between the groups in the two sub-cohorts (Training cohort (age mean and ratio female/male for all phenotypes: asymptomatic: age: 62.45, ratio=1; moderate: age: 60.13, ratio=1.14; severe: age: 57.18, ratio=0.86), validation cohort (age and ratio female/male for all phenotypes: asymptomatic: age: 52.82, ratio=1; moderate: age: 53.67, ratio=1.13; severe: age: 62, ratio=1)). The methylation data are available under the reference: (GEO accession: GSE191082).

### Biomarker identification for disease forms

Since data contains a lot of features (736,661), feature selection was performed in two steps, on the training group only. The scripts used for the following steps are available on Github (https://github.com/TAGC-ComplexDisease/biomarkersChagas). First, the delta beta (difference of beta means) was computed between the two phenotypes of interest. Only the CpGs having at least 10% methylation differences were retained. Then, a machine learning (ML) analysis was done in Python with Scikit-learn library. Four supervised ML methods were considered: decision tree, random forest, logistic regression and linear SVM (Support-Vector Machine, a linear classificator). For each method, recursive feature elimination (RFE) was performed, and the best model (best accuracy) with the minimal set of feature was selected using 10-time cross-validation. Finally, model parameters were optimized with a grid search to obtain the final prediction on the validation group.

## Results

### Symptomatic cardiac form prediction

**Figure 1:**
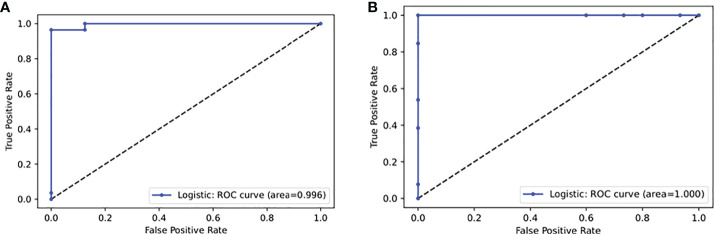
Receiver Operating Characteristic (ROC) curves produced by **(A)** linear SVM to predict CCC on 44 patients and **(B)** random forest to predict CCC stage on 28 patients.

**Figure 2:**
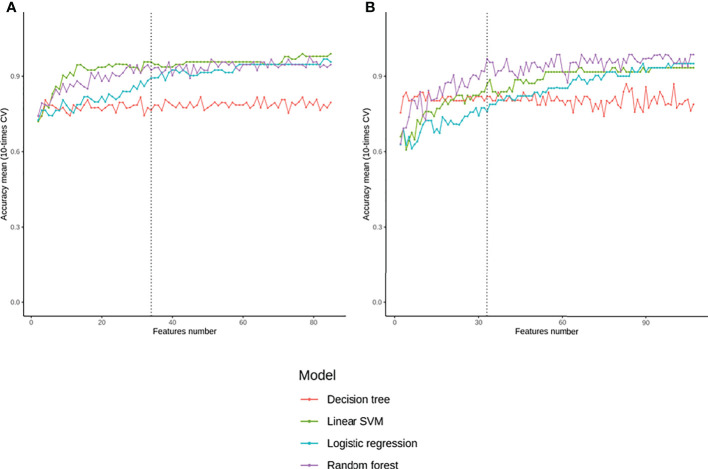
Evolution of the accuracy obtained with different machine learning models according to the number of top-explicative features chosen for **(A)** predict CCC or **(B)** predict CCC stage.

After feature selection based on delta beta values, 86 CpGs were selected. Among all the tested models, linear SVM seems to have the better prediction on training dataset with the minimal number of features (**Figure 2A**). According to this analysis, the SVM was trained with 35 features (**Supplementary Table 2**). The model parameters optimization was performed using a grid search where the L2 penalty varies between 0.01 and 10. Finally, with a L2 penalty of 1, 42 of 44 patients phenotype of the validation dataset were correctly predicted (accuracy = 0.95), with a sensitivity of 0.96 and a specificity of 0.94 (area under the curve: 0.996) (**Figure 1A**). Those 35 features are mainly located in the body of genes (n=20), or in intergenic regions (n=11). Particularly, 3 CpGs are located in LHX6, and 3 in POU6F2. All those genes are involved in biological process associated to Chagas disease: nervous system (LHX6, POU6F2, MDGA1, DISC1, PCSK9), immune system (ZMIZ, HLA-DRB1), Wnt pathway (DISC1), ion transport (KCNK15, PCSK9), striated muscles (SMYD3) or ATP metabolic process (ATP5S).

### Chagas cardiomyopathy stage prediction

After feature selection based on delta beta, 108 CpGs were selected. Among all the tested models, random forest seems to have the better prediction on training dataset with the minimal number of features (**Figure 2B**). According to this analysis, the SVM was trained with 33 features (**Supplementary Table 3**). The model parameters optimization was performed using a grid search where the number of estimators varies between 50 and 200. Finally, 150 estimators, 27 of 28 patients phenotype (accuracy = 0.96) of the validation dataset were correctly predicted, with a sensitivity of 1 and a specificity of 0.93 (area under the curve: 1) (**Figure 1B**). Those 33 features are mainly located in 18 intergenic regions. Other CpGs are located in 15 genes, and more precisely in 6 gene body and 7 promoter regions. Here, genes are involved in various biological processes, from ion transport (KCNC1, MFI2), actin filament (PACSIN1), generation of neurons (TNN, PACSIN1) or MAPK cascade (DUSP22). 2 CpGs are in common with those used as biomarker between ASY and CCC: cg24000535 (LOC101928909) and cg21873524 (intergenic).

## Discussion

Alterations of heart tissue DNA methylation profiles have been associated to the development of dilated cardiomyopathies (25, 26) and chagas diseases (21, 27). Recently, we have studied the DNA methylation in the blood of asymptomatic, moderate and severe CCC by hypothesizing that the blood data reflect the phenotype. We had found 12624 DMPs (Differentially Methylated Position) between asymptomatic and severe CCC blood samples and 6735 CpGs were found as DMPs between moderate and severe CCC.

In our study, based on machine learning approaches, we have identified 35 CCC-specific methylation markers. Those CpGs could distinguish controls (asymptomatic) to CCC from blood samples with 96% of sensitivity and 94% of specificity in independent validation sets. 3 of them (cg02872767, cg24540763, cg25134647) are also differentially methylated between asymptomatic and severe CCC in heart tissue (21). Similarly, 33 CpGs have been identified, allowing to predict the progression of this pathology (from moderate to severe CCC), with a sensitivity of 100% and a specificity of 93%. In conclusion, we identified two set of methylation markers potentially useful for Chagas disease diagnostic. The first one permit to discriminate patients with Chagas cardiomyopathy from asymptomatic patients, with 95% of precision. The second allows to predict Chagas cardiomyopathy severity stage, according to the heart ejection fraction rate, with 96% of precision.

Interestingly, most of these markers were not differentially in heart tissue of patients. The main message of this report is the finding that peripheral blood epigenetic marks are good markers of clinical form, implying that epigenetic events are closely related to CCC progression. These markers are highlighting the same biological processes that have been associated to the disease development such as ion transport, ATP metabolic process, immune system, Wnt system, nervous systems, striated muscles and actin filament. These findings are important as these are under-lighting biological pathways that will have to be targeted in drug design.

To propose large-scale reproducible biomarkers, a consensus Target Product Profile (TPP) has been developed for Chagas disease (28) stipulating that marker should be able to detect the effects of drug treatments, be detectable with limited resources and not vary according to the strain of the parasite. Given the high specificity of these assays, these methylation sites appear to be good candidates to decipher the pathogenic process, or to be used as blood biomarkers, and further studies will be necessary to potentially validate their possible use in the clinic, in accordance with the TPP consensus.

## Data availability statement

The datasets presented in this study can be found in online repositories. The names of the repository/repositories and accession number(s) can be found in the article/**Supplementary Material**.

## Ethics statement

The studies involving human participants were reviewed and approved by INSERM IRB University of Sao Paulo. The patients/participants provided their written informed consent to participate in this study.

## Author contributions

Study design: PBr, JK, LS, ECN, CC. Phenotype characterization: BI, CM, SS, CWP, BS, FD, MS, JAMN, AF, GDLP, PBu, HTL-W, AS, MM, MHH, ED, ACP, VRJ Experimental analysis: PBr, AFF, JPSN, PCT, LRPF, AK, DDSC, RCFZ, VOCR, RRA. Statistical analysis: PBr, LS, ECN, CC. Manuscript preparation: PBr, LS, ECN, CC.

## Funding

This work was supported by the Institut National de la Santé et de la Recherche Médicale (INSERM); the Aix-Marseille University (grant number: AMIDEX “International_2018” MITOMUTCHAGAS); the French Agency for Research (Agence Nationale de la Recherche-ANR (grant numbers: “Br-Fr-Chagas”, “landscardio”); the CNPq (Brazilian Council for Scientific and Technological Development); and the FAPESP (São Paulo State Research Funding Agency Brazil (grant numbers: 2013/50302-3, 2014/50890-5); the National Institutes of Health/USA (grant numbers: 2 P50 AI098461-02 and 2U19AI098461-06). This work was founded by the Inserm Cross-Cutting Project GOLD. This project has received funding from the Excellence Initiative of Aix-Marseille University - A*Midex a French “Investissements d’Avenir programme”- Institute MarMaRa AMX-19-IET-007. JN was a recipient of a MarMaRa fellowship. EC-N, JK, ALR and ECS are recipients of productivity awards by CNPq. The funders did not play any role in the study design, data collection and analysis, decision to publish, or preparation of the manuscript.

## Acknowledgments

Center de Calcul Intensif d'Aix-Marseille is acknowledged for granting access to its high performance computing resources.

## Conflict of interest

The authors declare that the research was conducted in the absence of any commercial or financial relationships that could be construed as a potential conflict of interest.

## Publisher’s note

All claims expressed in this article are solely those of the authors and do not necessarily represent those of their affiliated organizations, or those of the publisher, the editors and the reviewers. Any product that may be evaluated in this article, or claim that may be made by its manufacturer, is not guaranteed or endorsed by the publisher.
