# Supplementary material for: Blood DNA methylation marks discriminate Chagas cardiomyopathy disease clinical forms
Source: Front Immunol. 2022 Sep 29;13:1020572. doi: 10.3389/fimmu.2022.1020572 (PMC9558220; doi:10.3389/fimmu.2022.1020572)
Supplement: Supplementary file 1 [file Table_1.docx]

**Supplementary table 1.** Cohort included in the study.

| **ID** | **Phenotype** | **Sex** | **Age** | **EF** | **Cohorte** |
| --- | --- | --- | --- | --- | --- |
| **CTRL8** | ASY | F | - | 0.67 | Train |
| **CTRL9** | ASY | F | - | 0.59 | Train |
| **CTRL10** | ASY | F | - | 0.67 | Train |
| **CTRL11** | ASY | F | - | 0.65 | Test |
| **CTRL12** | ASY | M | - | 0.67 | Train |
| **CTRL13** | ASY | M | 54 | 0.66 | Train |
| **CTRL14** | ASY | M | 57 | 0.7 | Train |
| **CTRL15** | ASY | M | 61 | 0.68 | Train |
| **CTRL16** | ASY | F | - | 0.65 | Test |
| **CTRL17** | ASY | M | 57 | 0.66 | Test |
| **CTRL18** | ASY | F | 59 | 0.65 | Train |
| **CTRL19** | ASY | M | 68 | 0.67 | Train |
| **CTRL20** | ASY | F | 61 | 0.62 | Test |
| **CTRL21** | ASY | M | 62 | 0.66 | Train |
| **CTRL22** | ASY | M | 82 | 0.72 | Train |
| **CTRL23** | ASY | F | 43 | 0.62 | Test |
| **CTRL24** | ASY | F | - | 0.63 | Test |
| **CTRL25** | ASY | F | - | 0.67 | Train |
| **CTRL26** | ASY | M | - | 0.62 | Train |
| **CTRL27** | ASY | M | - | 0.68 | Test |
| **CTRL28** | ASY | M | - | 0.61 | Train |
| **CTRL29** | ASY | F | - | 0.75 | Train |
| **CTRL30** | ASY | F | - | 0.65 | Train |
| **CTRL31** | ASY | M | - | 0.65 | Test |
| **CTRL32** | ASY | F | - | 0.68 | Train |
| **CTRL33** | ASY | F | 70 | 0.7 | Train |
| **CTRL34** | ASY | F | 69 | 0.72 | Train |
| **CTRL35** | ASY | M | 61 | 0.78 | Test |
| **CTRL36** | ASY | F | 60 | 0.64 | Train |
| **CTRL37** | ASY | M | 61 | 0.56 | Train |
| **CTRL38** | ASY | F | 56 | 0.74 | Test |
| **CTRL39** | ASY | M | 44 | 0.55 | Train |
| **CTRL40** | ASY | M | 69 | 0.68 | Train |
| **CTRL41** | ASY | F | - | 0.62 | Train |
| **CTRL42** | ASY | F | 63 | 0.67 | Test |
| **CTRL43** | ASY | M | 49 | 0.76 | Test |
| **CTRL44** | ASY | M | 65 | 0.69 | Train |
| **CTRL45** | ASY | M | - | 0.77 | Train |
| **CTRL46** | ASY | F | 50 | 0.69 | Train |
| **CTRL47** | ASY | M | 31 | 0.55 | Test |
| **CTRL48** | ASY | F | 66 | 0.81 | Train |
| **CTRL49** | ASY | M | 42 | 0.65 | Test |
| **CTRL50** | ASY | M | 46 | 0.68 | Train |
| **CTRL51** | ASY | M | 83 | 0.65 | Train |
| **CTRL52** | ASY | F | 47 | 0.77 | Train |
| **CTRL53** | ASY | F | 76 | 0.46 | Train |
| **CTRL54** | ASY | M | 70 | 0.63 | Test |
| **CTRL55** | ASY | F | 48 | 0.71 | Test |
| **modCCC1** | modCCC | F | - | 0.68 | Test |
| **modCCC2** | modCCC | M | 43 | 0.52 | Test |
| **modCCC3** | modCCC | F | 59 | 0.68 | Train |
| **modCCC4** | modCCC | F | 81 | 0.77 | Train |
| **modCCC5** | modCCC | F | 35 | 0.67 | Train |
| **modCCC6** | modCCC | M | 37 | 0.53 | Test |
| **modCCC7** | modCCC | F | 39 | 0.62 | Train |
| **modCCC8** | modCCC | M | 51 | 0.56 | Test |
| **modCCC9** | modCCC | F | 66 | 0.68 | Train |
| **modCCC10** | modCCC | F | 68 | 0.45 | Train |
| **modCCC11** | modCCC | M | 49 | 0.63 | Train |
| **modCCC12** | modCCC | M | - | 0.45 | Train |
| **modCCC13** | modCCC | F | 70 | 0.67 | Train |
| **modCCC14** | modCCC | M | 54 | 0.56 | Train |
| **modCCC15** | modCCC | F | - | 0.58 | Test |
| **modCCC16** | modCCC | M | 54 | 0.46 | Train |
| **modCCC17** | modCCC | M | 65 | 0.3 | Train |
| **modCCC18** | modCCC | F | 61 | 0.61 | Test |
| **modCCC19** | modCCC | F | 61 | 0.57 | Train |
| **modCCC20** | modCCC | M | 61 | 0.78 | Train |
| **modCCC21** | modCCC | M | 50 | 0.69 | Train |
| **modCCC22** | modCCC | F | 65 | 0.66 | Train |
| **modCCC23** | modCCC | F | 52 | 0.47 | Test |
| **modCCC24** | modCCC | M | 85 | 0.53 | Test |
| **modCCC25** | modCCC | M | - | 0.58 | Train |
| **modCCC26** | modCCC | F | - | 0.68 | Train |
| **modCCC27** | modCCC | M | - | 0.52 | Train |
| **modCCC28** | modCCC | F | - | 0.55 | Train |
| **modCCC29** | modCCC | M | - | 0.7 | Train |
| **modCCC30** | modCCC | F | - | 0.66 | Train |
| **modCCC31** | modCCC | F | - | 0.49 | Train |
| **modCCC32** | modCCC | M | - | 0.69 | Train |
| **modCCC33** | modCCC | F | - | 0.65 | Test |
| **modCCC34** | modCCC | M | - | 0.58 | Test |
| **modCCC35** | modCCC | M | - | 0.5 | Test |
| **modCCC36** | modCCC | M | - | 0.5 | Train |
| **modCCC37** | modCCC | F | - | 0.66 | Train |
| **modCCC38** | modCCC | M | - | 0.65 | Train |
| **modCCC39** | modCCC | F | - | 0.56 | Train |
| **modCCC40** | modCCC | F | - | 0.71 | Train |
| **modCCC41** | modCCC | F | - | 0.48 | Test |
| **modCCC42** | modCCC | M | - | 0.47 | Train |
| **modCCC43** | modCCC | M | 78 | 0.7 | Test |
| **modCCC44** | modCCC | F | - | 0.64 | Test |
| **modCCC45** | modCCC | F | - | 0.67 | Test |
| **modCCC46** | modCCC | M | - | 0.7 | Train |
| **modCCC47** | modCCC | F | - | 0.64 | Train |
| **sevCCC11** | sevCCC | M | 54 | 0.25 | Train |
| **sevCCC12** | sevCCC | M | 65 | 0.3 | Train |
| **sevCCC13** | sevCCC | F | 63 | 0.26 | Train |
| **sevCCC14** | sevCCC | M | 84 | 0.25 | Train |
| **sevCCC15** | sevCCC | F | 49 | 0.39 | Train |
| **sevCCC16** | sevCCC | M | 67 | 0.26 | Test |
| **sevCCC17** | sevCCC | F | 56 | 0.25 | Train |
| **sevCCC18** | sevCCC | M | 63 | 0.28 | Train |
| **sevCCC19** | sevCCC | F | 52 | 0.28 | Test |
| **sevCCC20** | sevCCC | F | 60 | 0.25 | Test |
| **sevCCC21** | sevCCC | M | 57 | 0.27 | Test |
| **sevCCC22** | sevCCC | M | - | 0.2 | Train |
| **sevCCC23** | sevCCC | M | 48 | 0.2 | Train |
| **sevCCC24** | sevCCC | M | - | 0.32 | Test |
| **sevCCC25** | sevCCC | F | 52 | 0.33 | Train |
| **sevCCC26** | sevCCC | F | 50 | 0.3 | Test |
| **sevCCC27** | sevCCC | F | 88 | 0.31 | Test |
| **sevCCC28** | sevCCC | M | 52 | 0.19 | Train |
| **sevCCC29** | sevCCC | F | 28 | 0.3 | Train |
| **sevCCC30** | sevCCC | F | 66 | 0.22 | Test |
| **sevCCC31** | sevCCC | F | - | 0.22 | Train |
| **sevCCC32** | sevCCC | F | 45 | 0.3 | Train |
| **sevCCC33** | sevCCC | F | - | 0.36 | Train |
| **sevCCC34** | sevCCC | F | - | 0.23 | Train |
| **sevCCC35** | sevCCC | M | 53 | 0.31 | Test |
| **sevCCC36** | sevCCC | M | 65 | 0.34 | Train |
| **sevCCC37** | sevCCC | M | 71 | 0.22 | Test |
| **sevCCC38** | sevCCC | M | 52 | 0.29 | Train |
| **sevCCC39** | sevCCC | M | - | 0.26 | Test |
| **sevCCC40** | sevCCC | M | - | 0.26 | Train |
| **sevCCC41** | sevCCC | F | - | 0.24 | Train |
| **sevCCC42** | sevCCC | M | - | 0.18 | Train |
| **sevCCC43** | sevCCC | F | - | 0.33 | Test |
| **sevCCC44** | sevCCC | F | - | 0.27 | Train |
| **sevCCC45** | sevCCC | M | - | 0.28 | Train |
| **sevCCC46** | sevCCC | M | 47 | 0.23 | Train |
| **sevCCC47** | sevCCC | F | - | 0.25 | Train |
| **sevCCC48** | sevCCC | F | - | 0.14 | Train |
| **sevCCC49** | sevCCC | M | 67 | 0.25 | Train |
| **sevCCC50** | sevCCC | M | 65 | 0.25 | Test |
| **sevCCC51** | sevCCC | M | 73 | 0.22 | Train |
| **sevCCC52** | sevCCC | F | - | 0.34 | Train |
| **sevCCC53** | sevCCC | F | - | 0.22 | Train |

EF : Ejection Fraction
