# Supplementary material for: Blood DNA methylation marks discriminate Chagas cardiomyopathy disease clinical forms
Source: Front Immunol. 2022 Sep 29;13:1020572. doi: 10.3389/fimmu.2022.1020572 (PMC9558220; doi:10.3389/fimmu.2022.1020572)
Supplement: Supplementary file 2 [file Table_2.docx]

**Supplementary table 2.** List of CpGs of interest useful to discriminate CCC patients from asymptomatic subjects.

| **CpG ID** | **Chromosome** | **Position** | **Gene** | **Localization** |
| --- | --- | --- | --- | --- |
| cg20245116 | 1 | 55522013 | PCSK9 | Body |
| cg21109666 | 1 | 232007841 | DISC1 | Body |
| cg04798314 | 1 | 246668601 | SMYD3 | Body |
| cg12880874 | 2 | 2942535 | LINC01250 | Body |
| cg09399716 | 2 | 46890238 |  | IGR |
| cg13883027 | 2 | 62892060 |  | IGR |
| cg21873524 | 4 | 190942744 |  | IGR |
| cg10788837 | 5 | 133089737 |  | IGR |
| cg02891314 | 5 | 179741120 | GFPT2 | Body |
| cg13746813 | 6 | 14911904 |  | IGR |
| cg08269402 | 6 | 32549631 | HLA-DRB1 | Body |
| cg02267270 | 6 | 37616410 | MDGA1 | Body |
| cg25069157 | 6 | 44102572 | TMEM63B | Body |
| cg06696596 | 6 | 122391246 |  | IGR |
| cg22772380 | 7 | 6747037 | ZNF12 | TSS1500 |
| cg06405219 | 7 | 30219135 |  | IGR |
| cg15212455 | 7 | 39170539 | POU6F2 | Body |
| cg08835956 | 7 | 39171034 | POU6F2 | Body |
| cg21665744 | 7 | 39171113 | POU6F2 | Body |
| cg27048067 | 8 | 674560 | ERICH1 | Body |
| cg04282082 | 9 | 124988720 | LHX6 | Body |
| cg21469772 | 9 | 124989294 | LHX6 | Body |
| cg03363289 | 9 | 124990165 | LHX6 | Body |
| cg06878111 | 10 | 9999498 |  | IGR |
| cg20744163 | 10 | 80999841 | ZMIZ1 | Body |
| cg25134647 | 12 | 25454990 |  | IGR |
| cg24540763 | 12 | 122377170 | WDR66 | Body |
| cg10788750 | 13 | 78973425 | RNF219-AS1 | Body |
| cg17797229 | 13 | 110522297 |  | IGR |
| cg25787588 | 14 | 50784952 | ATP5S | Body |
| cg24000535 | 14 | 91110600 | LOC101928909 | Body |
| cg12036633 | 15 | 63758958 |  | IGR |
| cg18128914 | 15 | 74244249 | LOXL1 | 3'UTR |
| cg02872767 | 19 | 1525453 | PLK5P | Body |
| cg25301532 | 20 | 43378953 | KCNK15 | Body |

IGR : intergenic region

TSS200 : region from Transcription start site (TSS) to − 200 nt upstream of TSS

TSS1500 : region from Transcription start site (TSS) to − 1500 nt upstream of TSS
