# Supplementary material for: Blood DNA methylation marks discriminate Chagas cardiomyopathy disease clinical forms
Source: Front Immunol. 2022 Sep 29;13:1020572. doi: 10.3389/fimmu.2022.1020572 (PMC9558220; doi:10.3389/fimmu.2022.1020572)
Supplement: Supplementary file 3 [file Table_3.docx]

**Supplementary table 3.** List of CpGs of interest useful to discriminate severe CCC patients from moderate CCC patients.

| **CpG ID** | **Chromosome** | **Position** | **Gene** | **Localization** |
| --- | --- | --- | --- | --- |
| cg23658987 | 1 | 175047784 | TNN | Body |
| cg13263947 | 2 | 191443609 |  | IGR |
| cg07030646 | 3 | 30565915 |  | IGR |
| cg02931642 | 3 | 184320734 |  | IGR |
| cg18522231 | 3 | 196705629 |  | IGR |
| cg15727583 | 3 | 196757701 | MFI2 | TSS1500 |
| cg21873524 | 4 | 190942744 |  | IGR |
| cg06961054 | 5 | 56204405 | SETD9 | TSS1500 |
| cg04481923 | 5 | 135416205 | MIR886 | Body |
| cg18110333 | 6 | 292329 | DUSP22 | 1stExon |
| cg25107000 | 6 | 31275643 |  | IGR |
| cg02032966 | 6 | 34482493 | PACSIN1 | TSS200 |
| cg06864789 | 6 | 139012992 |  | IGR |
| cg18136963 | 6 | 139013146 |  | IGR |
| cg17240725 | 7 | 73149356 | WBSCR26 | TSS200 |
| cg03327325 | 10 | 43846574 |  | IGR |
| cg05871802 | 11 | 17788335 | KCNC1 | Body |
| cg07136909 | 11 | 71278894 |  | IGR |
| cg16098618 | 11 | 76368494 |  | IGR |
| cg11526635 | 11 | 117988809 | TMPRSS4 | 3'UTR |
| cg11635454 | 11 | 126173444 | DCPS | TSS1500 |
| cg06067394 | 11 | 133789110 | IGSF9B | Body |
| cg20141578 | 12 | 12225262 | BCL2L14 | 5'UTR |
| cg13422161 | 12 | 52773842 | KRT84 | Body |
| cg24000535 | 14 | 91110600 | LOC101928909 | Body |
| cg23758822 | 17 | 41437982 |  | IGR |
| cg06875181 | 17 | 76649257 |  | IGR |
| cg13023205 | 17 | 81060243 |  | IGR |
| cg20269954 | 18 | 27037507 |  | IGR |
| cg27105123 | 19 | 55582635 |  | IGR |
| cg22459517 | 19 | 55587193 | EPS8L1 | TSS200 |
| cg08564027 | 20 | 61660810 |  | IGR |
| cg01584094 | 21 | 45254457 |  | IGR |

IGR : intergenic region

TSS200 : region from Transcription start site (TSS) to − 200 nt upstream of TSS

TSS1500 : region from Transcription start site (TSS) to − 1500 nt upstream of TSS
